# Determining Upper Limit of Alanine Aminotransferase in Iranian Cohort Population Using Ultrasound Screening for Liver Diseases

**DOI:** 10.34172/mejdd.2025.426

**Published:** 2025-07-30

**Authors:** Behnam Ahmadi, Farhad Naleini, Mehdi Moradinazar, Bita Anvari

**Affiliations:** ^1^Radiology Department of Imam Reza Hospital, School of Medicine, Kermanshah University of Medical Sciences, Kermanshah, Iran; ^2^Social Development and Health Promotion Research center, Health Policy and Promotion research center, Kermanshah University of Medical Sciences, Kermanshah, Iran; ^3^Internal Medicine Department, Imam Khomeini Hospital, School of Medicine, Kermanshah University of Medical Sciences, Kermanshah, Iran

**Keywords:** ALT, SGPT, Liver transaminases, Ultra-sonography, MASLD, Fatty liver, Sensitivity

## Abstract

**Background:**

Alanine aminotransferase (ALT) has a variable normal range according to race and ethnicity. So, the upper normal level of ALT localized for the Iranian population was determined in the Ravansar Non-Communicable Disease (RaNCD) cohort population by re-evaluation of high-risk people.

**Methods:**

A cohort population with normal ALT results based on the current kit was checked for a history of liver diseases. After excluding them, the remaining population was included in the distribution diagram of individuals with apparently healthy livers. Participants whose ALT values were in the 90th to 100th percentile were re-evaluated by ultrasonography (US) and a checklist of liver disease. Patients identified as having liver disease or those with other abnormal liver enzymes were excluded, and the 95th percentile was extracted from the distribution diagram of the remaining population.

**Results:**

After excluding liver disease, among 8046 participants of RaNCD, US and re-evaluation were performed in 543 high-risk individuals. Liver disease was diagnosed in 74.6% by US. The most common liver disease was metabolic dysfunction-associated steatotic liver disease (MASLD), accounting for 69.7%. Grade 2 or 3 of MASLD was found in 23.2%. After excluding patients with abnormal liver enzymes and liver disease, the 95th percentile of ALT was 29 U/L in women (sensitivity: 53%, specificity: 82%) and 36 U/L in men (sensitivity: 28%, specificity: 90%).

**Conclusion:**

The calculated 95th percentile was lower than the routine cut-off value of the current kit in both sexes. Generalizability is a significant advantage of our results, provided by the lack of exclusion of patients with metabolic risk factors and the use of US to exclude MASLD.

## Introduction

 Alanine aminotransferase (ALT or SGPT) is a biomarker of hepatocyte damage, and clinicians typically request it as part of the initial, inexpensive laboratory evaluation for screening liver diseases, in addition to other liver enzymes. The current upper limit of normal (ULN) ALT, which is widely used in standard laboratory Kits, was computed initially based on the ALT measured in blood donors, which is mostly higher than the result of recent studies.^1-3^ This ULN is varied by ethnicity, age, sex, and indices of metabolic syndrome, and many studies computed its level in their own countries, like Koreans, Chinese, Indians, Turks, Americans, Vietnamese, and Taiwanese.^4-7^ According to a systematic review in 2020, there is diversity in the reported ALT levels in most studies. They recommended some adjustments to create a more effective tool for screening hepatocyte injury, particularly metabolic dysfunction-associated steatotic liver disease (MASLD).^8,9^ On the other hand, many large studies showed the association between mortality and ALT level even in the present normal range ( > 20 IU).^10^

 In five previous studies in Iran, the ULN ALT was determined in different populations: Two studies in blood donors^11,12^ and three studies on the normal population. In the normal population, one of the studies was conducted in the old population in Kalaleh,^13^ the secondone by Jamali et al in Golestan that emphasized on different level of ULN according to metabolic risk factors especially diabetes and body mass index (BMI)^14^ and the third by Akhondi-Meybodi et al in Yazd who calculated ULN ALT in population with and without diabetes.^15^ All previous studies excluded patients with definite liver disease and included all uninvestigated patients who had abnormal ALT levels by the present standard Kits, except for those with metabolic risk factors.

 This different pattern of population sampling strongly affects the ULN range computed. The researchers’ effort to consider MASLD as the most prevalent etiology of transaminitis was focused on excluding obese, hyperlipidemic, or diabetic patients, or stratifying different ULN levels in the high-risk population with indices of metabolic syndrome. This sampling method could exclude many target populations that need the calculated result of the ALT cut-off for transaminase interpretation.^12,15^

 So in this study, instead of excluding all patients with metabolic risk factors, we excluded only patients with MASLD, and we performed it by case finding of patients with MASLD by ultrasonography (US). Identification of hepatic steatosis with an invasive gold standard procedure like liver biopsy cannot be performed especially in the normal population. So, hepatic ultrasound with low cost, as a non-invasive procedure to screen for hepatic steatosis or even cirrhosis, was performed in this study. Fatty liver grading system in the US has been established previously^16^ and its accuracy in comparison to histology has been approved, especially in moderate-severe fatty liver disease.^17^

 Our study was conducted on a cohort population in Ravansar, situated in the Kermanshah province. The Ravansar Non-Communicable Disease (RaNCD) cohort study is a subset of the larger Prospective Epidemiological Research Studies in IrAN (PERSIAN) cohort, which spans across 21 centers.^18^ Its complete protocol guidance has been published as “Cohort profile” in 2019.^19^ So this sample provided the appropriate data generalizable to the community, especially in the west region of Iran, and the upper limit of ALT was computed as the 95^th^ percentile with sensitivity and specificity analysis.

## Materials and Methods

###  Study Design and Population 

 The study was conducted in September 2022 on individuals who participated in the RaNCD as part of the PERSIAN cohort.

 The research protocol contained two phases. In the first phase, the gathered data were reviewed, and participants who had at least one result of ALT level were selected. The primary goal was to exclude all patients who had any liver diseases or hepatic complications of other diseases involving the liver, which may disturb liver enzymes (like metastatic cancers with hepatic involvement).

 Afterward, the determination of the 95^th^ percentile of ALT in the remaining normal population without recognized liver disease was done. This designation prepared an appropriate normal population as the sample without exclusion of many diseases such as diabetes, hyperlipidemia, or obesity, which are high-risk groups for hepatic steatosis but have no evidence of liver steatosis, at least as indicated by the usual non-invasive liver screening tests. This design could increase the generalizability of the upper limit of ALT defined in this research to the entire normal population without recognized liver disease, including subsets of diabetic, overweight, or hyperlipidemic patients.

###  Inclusion and Exclusion Criteria 

 Primary data from participants who had at least one result with an ALT level were selected. Patients with abnormal levels (more than 41 U/L) were excluded, as determined by the ALAT Kit provided by Pars Azmoun laboratory company, due to suspected obscure liver disease. Also initial histories in addition to outcomes of RaNCD were evaluated to exclude patients with any history of liver diseases including fatty liver disease (MASLD), cirrhosis, hepatitis B or C, alcoholic liver disease, Wilson disease, hemochromatosis, autoimmune or drug induced hepatitis and cancers including metastatic liver involvement, hepatocellular carcinoma or hematological malignancies. All of these patients were excluded, and the normal-appearing population was used to create a distribution diagram of ALT.

 Primary analysis was done at this step to select the best group for diagnostic intervention in the 2^nd^ phase. The diagram showed a shift to the right (to the upper border of ALT enzyme level) and we concluded abnormal population may be the etiology of this shift even in the present normal range of Kit and they could be those patients with obscure liver diseases especially MASLD due to high prevalence of metabolic syndrome in the recent years.

###  Study Design and Population in the 2^nd^ Phase

 The population selected as the high-risk group for diagnostic intervention was defined as those with ALT levels between the 90th and maximum normal ALT levels (below 41 U/L). Most of this high-risk group population was men. To balance the sex ratio following the application of the 1st phase exclusion criteria, an equal number of men and women were selected to participate in the diagnostic screening for liver disease.

###  Data Collection and Measurements in the 2^nd^ Phase

 Regarding the diagram shift, in this phase, the high-risk group for liver disease was selected for a diagnostic intervention using liver sonography and a further review of the history for liver disease, as mentioned in the exclusion criteria. Other risk factors like diabetes, hyperlipidemia, hepatotoxic drug consumption, congestive heart failure or corpulmonale, celiac disease, inflammatory bowel disease, thyroid disease, or other liver-related diseases were all gathered by a checklist. This checklist consisted of US data, including focal fatty infiltration or sparing, grading of hepatic steatosis, hemangioma, cyst, mass, cirrhosis, hepatosplenomegaly, or other incidental sonographic findings related to liver disease. The US evaluation of hepatic steatosis was defined based on a qualitative visual assessment of hepatic echogenicity, comparing the echogenicity of the liver parenchyma with that of the cortex of the right kidney, evaluating echo penetration into the deep portion of the liver, and determining the clarity of the diaphragm and the echogenicity of the wall of the intrahepatic portal veins. Sonography was performed using a Samsung WS80A machine at the Ravansar clinic.

###  Exclusion Criteria of Ultra-sonographic and Other Paraclinical Measurements

 After the 2^nd^ phase, patients who were diagnosed with liver disease (according to the exclusion criteria) or those with US findings of steatosis grade 2 or 3, hemangioma, cirrhosis, liver cyst, mass, or hepatomegaly in sonography were excluded as involved patients with liver disease. Hepatomegaly was defined by liver span in US greater than 160 mm.

 Also, other paraclinical laboratory findings in the RaNCD cohort which were indexes of liver disease or were related to liver damage including elevated gamma glutamyl transferase (γGT), aspartate amino transferase (AST) or even alkaline phosphatase (ALP) were checked and all patients with any abnormal liver enzyme were excluded from the normal population as having obscure liver disease. Abnormal level was defined as AST ≥ 41 U/L, γGT ≥ 41 U/L, Alk ph. ≥ 306 IU/L according to ALAT Kit.

###  Statistical Analysis

 Central tendency measures were used to describe quantitative variables, while for qualitative variables, frequency and percentage were employed. In this study, the 5^th^ and 95^th^ percentiles of ALT were calculated for the remaining normal population after screening for high-risk groups for liver diseases. The 95^th^ percentile was introduced as the upper limit of ALT in males and females separately. The evaluation of the model’s performance was conducted using several metrics, including the area under the receiver operating characteristic (ROC) curve (AUC), sensitivity, and specificity. A two-sided *P* value < 0.05 was considered significant, and all analyses were conducted using STATA software, version 14.

## Results

 The RaNCD cohort was conducted on 10,047 participants, whose demographic features, such as age, sex, BMI, diet, and physical activity, as well as the prevalence of non-communicable diseases, including hypertension, diabetes, dyslipidemia, and a previous history of fatty liver disease, were published in the “Cohort profile” in 2019.^19^

 Regarding their liver enzymes, 64 participants had normal ALT results, and 929 patients had abnormal ALT levels ( ≥ 41 U/L). Among the remainder, 1036 patients had history of fatty liver disease (renamed as MASLD), 12 patients had hepatitis B and three hepatitis C, four patients had cirrhosis including one patient with chronic hepatitis B infection, the 2^nd^ one with sclerosing cholangitis, 3^rd^ patient with metabolic dysfunction associated steatohepatitis (MASH) with diabetes and lichen planus and 4^th^ patient with extrahepatic biliary stricture following cholecystectomy. Three patienst with cirrhosis received liver transplants, and the patient with hepatitis B is still receiving tenofovir.

 Eighty-three patients had different types of cancers, including one non-Hodgkin lymphoma and one leukemia. One patient had cholangiocarcinoma, another one was involved by colon cancer with hepatic metastasis, and two patients had liver cancer, one of them had metastatic disease; all expired during the 7-year serial follow-up of RaNCD cohort study.

 Alcohol ingestion with a variable amount was found in 487 participants, whose ALT was abnormal in 81 patients (16.6%).

 Hemochromatosis, Wilson’s disease, autoimmune hepatitis, and celiac disease were not identified by the outcome search using ICD-10-related codes.

 A total of 2000 patients were excluded to create a group of normal population without liver diseases or other diseases with hepatic complications. 8046 participants, including 3739 men (46.5%) and 4307 women (53.5%), remained. In this group, the mean ALT was 21.2 ± 7.3 U/L (3.1-41) and the 95th percentile was 37.7 U/L for men and 31.3 U/L for women.

**Figure 1:**
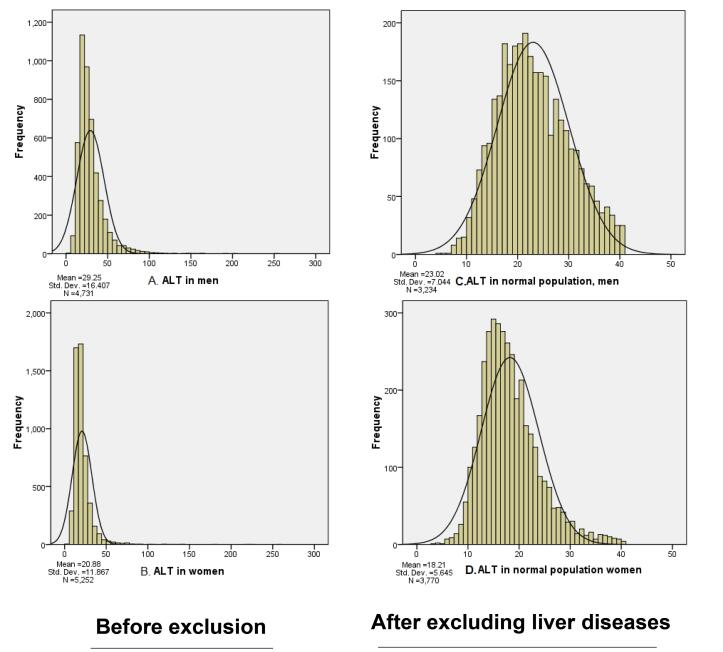
ALT level distribution was analyzed according to sex in the All RaNCD cohort population (diagram A in men, B in women). After deletion of patients with liver disease and abnormal liver aminotransferases γGT and alkaline phosphatase, finally the rest of the normal population were analyzed in diagrams C and D. RaNCD: Ravansar Non-Communicable Diseases

 A right-sided shift in the distribution diagram of ALT was shown according to sex in Figure 1 (Diagram A and B).

**Figure 2:**
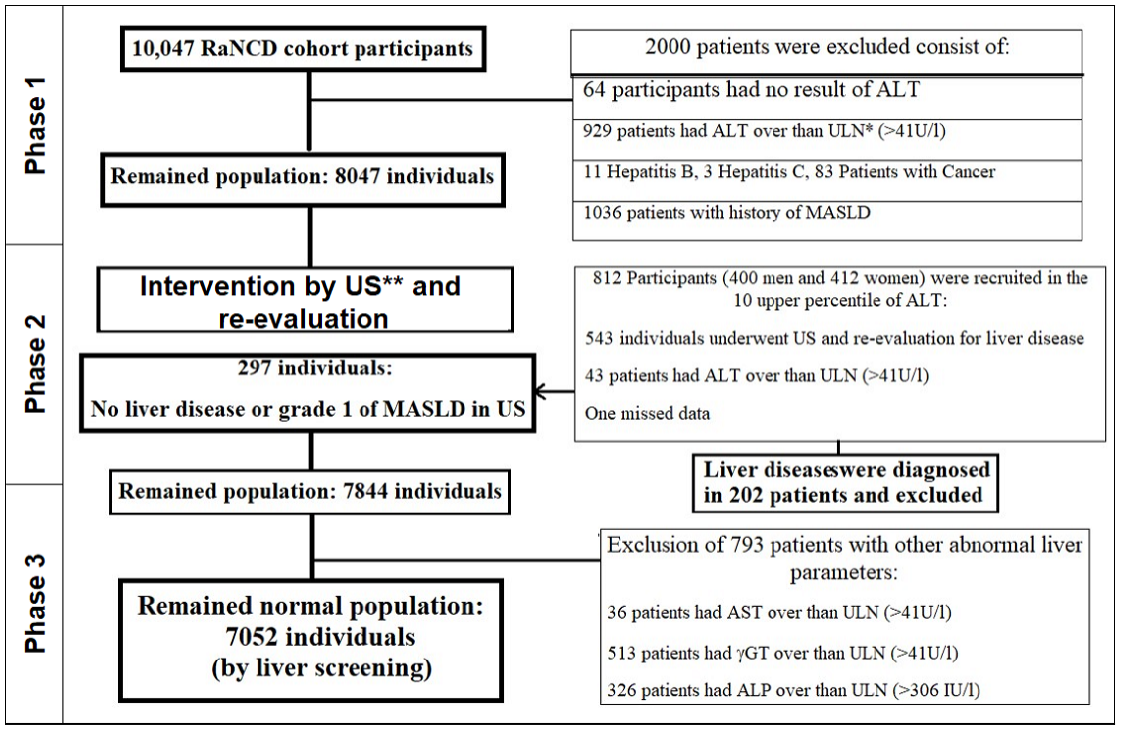
Stepwise investigation for liver involvement in the cohort of RaNCD to purify the normal population. ULN: Upper limit of normal range according to the standard kit. ** US: Ultrasonography

 Then, in the second phase, 812 individuals with ALT values between the 90th percentile and the ULN ( < 41 U/L) were selected to participate in the diagnostic screening for liver disease (flowchart in Figure 2).

 Due to the dominance of male sex in these upper percentiles and to balance the sex, 412 men and 400 women were recalled by telephone to complete the consent form and participate in the diagnostic screening of liver disease by US and another re-evaluation of liver disease with a checklist.

 Finally, 543 individuals participated in this phase. Again, 43 patients had abnormal ALT. One of the results of US and data of re-evaluation was missed, and other findings, including US findings, demographic features, and the ranges of their liver parameters, are summarized in Tables 1-3.

**Table 1:** Results of sonographic findings in liver screening (A) and confirmed cases of liver disease with respect to inclusion or exclusion of grade 1 of fatty liver diseases before and after excluding patients with abnormal ALT (B)

**A. Ultra-sonographic findings**			
**US findings marked as liver outcomes**	**No. (%)**	**Other US findings**	**No. (%)**
Grade 1	244 (44.9)	Cholelithiasis (Single)	15 (2.8)
Grade 2	119 (21.9)	Cholelithiasis (Multiple or sludge)	14 (2.6)
Grade 3	7 (1.3)	Polyp of the gall bladder (single)	2 (0.4)
Liver cyst	3 (0.6)	Multiple polyps of the gall bladder	1 (0.2)
Liver cysts (Bilateral Polycystic Kidney Disease)	1 (0.2)	Adenomyomatosis of the gall bladder	2 (0.4)
Liver hemangiomas	6 (1.1)	Ectopic gall bladder	1 (0.2)
Hepatomegaly	90 (16.6)	Hematoma in the bed of cholecystectomy	1 (0.2)
Hepatosplenomegaly	2 (0.4)	Von Myenberg complex^a^	1 (0.2)
Cirrhosis (HBs Ag +)	2 (0.4)^b^	Hepatic granuloma	1 (0.2)
Liver mass	2 (0.4)	Calcified hepatic granuloma	2(0.4)
Focal fatty infiltration	3 (0.6)	Absent right kidney	1 (0.2)
Focal fatty sparing	86 (15.8)	Reidel lobe (liver)	2 (0.4)
No fatty liver	172 (31.7)	Splenomegaly	11 (2)
Total	542	Multiple confluent calcifications in the spleen	1 (0.2)
**B. **	**Total population screened (Total:542)**	**Screened population after excluding patients with abnormal ALT (499 patients)**	
**Presence of liver outcome**	**No. (%)**	**No. (%)**	
Liver disease (including grades 2&3 of fatty liver)	224 (41.3)	202 (40.4)	
Liver disease (including grade 1-3 of fatty liver)	411 (75.7)	373 (74.6)	
No liver disease (even fatty liver grade 1)	131 (24.1)	126 (25.2)	
No liver disease (including fatty liver grade 1)	318 (58.6)	297 (59.4)	

^a^Hamartoma in Intra hepatic canaliculi. ^b^One of them had a lot of collateral veins (portal hypertension).

**Table 2:** Demographic features of both populations screened for liver disease in the 2^nd^ phase (ALT between percentiles of 90^th^-100) and finally remained in the normal population

		** Screened population (n=543)**			**Total remained normal population (N=7052)**		
		No. (%)			No. (%)		
Sex	Male	280 (51.6)			3248 (46.06)		
	Female	263 (48.4)			3804 (53.94)		
Diabetes		85 (15.7%)			558 (7.91)		
Prediabetes		32 (5.9)					
Hyperlipidemia (by history)		164 (30.4)					
Hyperlipidemia in laboratory tests		278 (51.2)			3113(44.15)		
Hypertension		131 (24.1)			1484 (21.04)		
Cardiac disease		33 (6.1)			260 (3.69)		
Hypothyroidism		32 (5.9)			226 (3.21)		
Fatty liver disease (by history)		70 (12.9)			-		
Thrombocytopenia		20 (3.7)			155(2.2)		
Drug history	Statin	870 (12.34)			870 (12.34)		
	Prednisolone	188 (0.03)			188 (0.03)		
	Methotrexate	42 (0.01)			42 (0.01)		
		**Mean (SD)**	**Min.**	**Max.**	**Mean (SD)**	**Min.**	**Max.**
Age		46.9 (7.7)	35	65	47.19 (8.3)	35	65
BMI		27 (4.2)	15.1	52.8	27.51	12.5	52.8
WHR		0.95 (0.5)	0.8	1.18	0.94	0.69	1.5

BMI: Body mass index, WHR: Waist hip ratio.

**Table 3:** Liver parameters in the high-risk population with ALT between the 90^th^ percentile and the upper limit of normal according to the grading of fatty liver in ultrasonography (US)

**Grading of fatty liver in the US**		SGPT*	SGOT**	**γGT**	**Alkaline phosphatase**
No fatty liver	No. of participants	172			
	Min.	11.2	9	6	83..4
	Max	68.6	57	141	444
Grade 1	No.	244			
	Min.	11.2	11.7	8.3	67.9
	Max	65	47.1	248	469.6
Grade 2	No.	119			
	Min.	19.1	15.8	13.5	94.6
	Max	66.5	40.8	104	365.4
Grade 3	No.	7			
	Min.	26.7	19	18.5	126.1
	Max	39.9	27.9	78.9	324.5
Total	No.	543			
	Min.	11.2	9	6	67.9
	Max	68.6	57	248	469.6
*P* value		0.2	0.3	0.1	0.1

*SGPT: ALT, **SGOT: AST

 As defined in exclusion criteria of US findings, patients who had fatty liver grade 2 or 3, cirrhosis, hepatomegaly with or without splenomegaly, liver cyst (including one patient with multiple bilateral renal cysts who was diagnosed as polycystic kidney disease), mass, haemangioma or even history of fatty liver were excluded from the normal population. Fatty liver grade 1 was excluded from the liver diseases due to operator dependency, low sensitivity, and high frequency in the normal population (Table 1, Panel B). Overall, 202 patients were identified as having liver disease involvement in the 2nd phase, and 297 normal individuals remained in the distribution diagram of the normal population. Distribution of ALT according to involvement by liver disease (after 2nd phase) and its categorization is summarized in Figure S1.

 Diabetes, dyslipidemia, and BMI have a significant association with fatty liver in the US (*P* < 0.001). Age had an inverse correlation with fatty liver, especially after the age of 45 (*P* < 0.001), and male sex had an insignificant association with fatty liver in the US (*P* = 0.05).

 In the screened population, diabetes and prediabetes were found in 14.2% of patients with liver disease in comparison to 7.2% among healthy people without liver disease who were in the upper 10th percentile of ALT (*P* < 0.001).

 In all the diabetic patients, the 95^th^ percentile of ALT was 32.5 IU/L in men and 29.5 IU/L in women after excluding patients with liver disease.

 Both ALT and γGT had no significant association with fatty liver in the US (*P* = 0.2 for ALT and 0.1 for γGT). Although ALT had low sensitivity to screen liver disease, γGT could be a better predictor of fatty liver disease (Table S1, Supplementary file 1)

 Available liver parameters in cohort profile, consist of γGT, AST and alkaline phosphatase were reviewed at this step and again 793 patients were recognized as obscure liver disease only by these abnormal findings (Figure 2). So this is the last exclusion criteria to purified normal population used to determine the percentile of 95^th^ and 7052 normal individuals without recognized liver disease were remained. Their demographic features are summarized in Table 2, and the 95th percentile for ALT was 36.1 (36) U/L in men and 28.8 (29) in women (Table 4).

**Table 4:** Percentiles of 5^th^ and 95^th^ of liver parameters in the normal cohort population (after excluding liver disease)

	**Sex**	**No. of pupulation**	**Min**	**Max**	**Mean (SD)**	**5**^th^ ** percentile**	**50**^th^ **percentile**	**95**^th^ ** percentile**
SGPT	male	3248	4	40.9	23.1 (7.1)	12.5	22.3	36.1
	female	3804	3.1	40.7	18.2 (5.6)	10.6	17.3	28.8
	Total	7052	3.1	40.9	20.4 (6.8)	11.2	19.3	33.8
SGOT	male	3248	9.5	40.7	20.7 (4.6)	14.2	20.1	29
	female	3804	7.5	40.1	18.4 (4.4)	12.5	17.7	26.5
	Total	7052	7.5	40.7	19.4 (4.6)	13	18.9	28
γGT	male	3248	4.9	40.9	21 (7.4)	11	20	35.6
	female	3804	1	40.9	17.1 (7)	8.7	15.4	31.7
	Total	7052	1	40.9	18.9 (7.4)	9.3	17.5	33.9
Alkaline phosphatase	male	3248	29	305.8	189.9 (42.9)	125.7	187.2	267.2
	female	3804	28.1	305.4	184.2 (48)	113	179	275
	Total	7052	28.1	305.8	186.8 (45.8)	118	183.1	271.4

 The effect of excluding patients with liver disease is evident in the distribution diagram of ALT levels in the normal population, according to the presence of liver disease, as shown in Figure 1 (Diagrams C and D).

**Figure 3:**
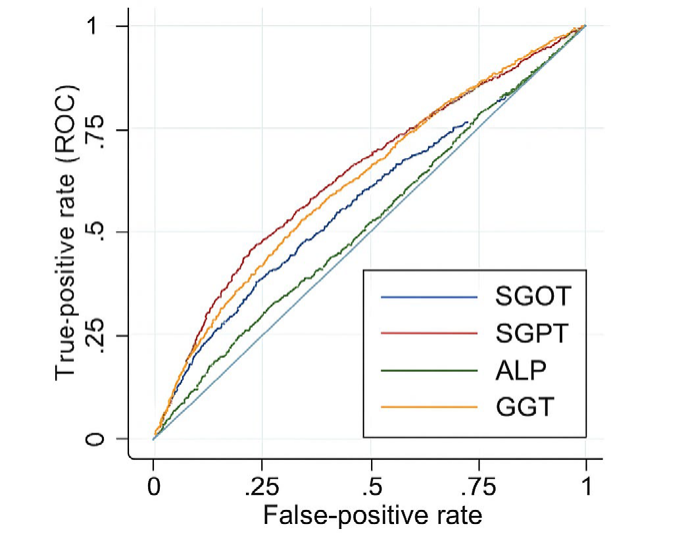
Area under the Receiver Operating Characteristic (AUROC) curve, sensitivity and specificity analysis for SGOT, SGPT, γGT and ALP in RaNCD cohort population to detect liver disease. GGT: Gamma glutamyl transferase

 In addition to determining the 95th percentile of ALT, its sensitivity, specificity, and positive and negative likelihood ratios for detecting liver disease were calculated (see Table S2 in the online Supplementary file 1). The best estimated level of ALT was calculated using the area under the receiver operating characteristic (AUROC) analysis to achieve the best specificity and sensitivity concurrently (Figure 3).

 Despite improving sensitivity (but with lower specifity), still newer cut off has not enough sensitivity (60%) for early detection of liver disease and this finding is more prominent for women whose most sensitive estimated cut off seems to be significantly lower than the calculated cut off value (19.7 U/L vs 28.8 U/L).

## Discussion

 Determination of the best ULN ALT to achieve both enough sensetivity to detect hepatocyte damage in the initial phase in addition to acceptable specifity especially in a sample of normal population by excluding patients with liver disease and those with uninvestigated abnormal liver parameters showed the best ULN ALT are 29 U/L in women and 36 U/L in men (by 95^th^ percentile of ALT).

 These upper limits have higher specificity in the presence of low sensitivity, in comparison to the best calculated values by AUROC analysis (29 U/L in men and 20 U/L in women), which have higher but not sufficiently acceptable sensitivity (71.7% in men and 60.7% in women) concomitant with lower specificity. Determination of ULN ALT by using the 95^th^ percentile could be seen in many articles.^9^ ALT as a screening tool for liver injury needs to be sensitive enough, and determining a more sensitive cut-off for early detection of liver disease would decrease its specificity in our study. So we recommend using the 95^th^ percentile as ULN to detect high-risk patients to determine the necessity of more liver evaluation due to its significant specificity. However, ALT as a tool for screening liver disease has low sensitivity, especially in our data, for example, in comparison to Chinese results.^20^ They chose cut points of AURAC analysis due to acceptable sensitivity (89% for 22 IU). We believed that physicians should be informed about the lower cut off values like 20 U/L in women and 29 U/L in men (by AUROC analysis) with the expense of lower specifity to increase earlier detection of minor liver problem however ignoring the specifity and determining ULN recommended by AUROC instead of 95^th^ percentile could detect minor liver problem however lead the physicians to perform unnecessary and expensive evaluations without acceptable benefit for the patients.

 Along with the development of technology and the availability of expensive and sometimes complex modalities, such as MRI or Fibroscan, in recent years, which are more accurate, US, as an available and non-invasive procedure, seems to be overlooked, especially due to inconsistencies about its accuracy.^21^ However, physicians should be informed about the proven sensitivity and specificity of US (84.8% and 93.6%, respectively) in moderate-severe fatty liver disease, in comparison to liver biopsy, as shown in a systematic review. They should also be aware of its low accuracy only in grade 1 fatty liver disease by US.^17^ This is the background for excluding grade 1 fatty liver disease (as assessed by US) from patients with MASLD as a type of liver disease in our study. This study was the first research in Iran to determine the ULN ALT, which used the US for diagnosis to determine the ALT cut-off. In large studies from Korea, India, and Taiwan, US was performed to diagnose MASLD as an exclusion criterion; however, its staging and pattern of definition and exclusion were not reported.^9,22^

 Prevalence of fatty liver by US in our results was 68.1% and grade 1 of fatty liver disease was found in 44.9% of participants. This prevalence was significantly higher compared to the majority of articles^23^ and may be due to the high prevalence of grade 1 fatty liver disease, which is partly operator-dependent and was not reported by many researchers. Another explanation is the pattern of our sampling in the high-risk group population, whose ALT values were in the maximum 10th percentile of the normal range, in order to detect patients with liver disease and MASLD.

 The concern about the occuracy of the standard kit cut-off value of liver transaminases developed mostly during the last two decades. The primary measurement of ULN ALT was performed in blood donors from the sixth to seventh decades of the 20^th^ century.^1,24,25^ Until now, large population studies reported new ULN and emphasized the underdetection of liver disease by current ULN, especially in the normal population.^20^ Our findings, similar to those of these research studies, showed lower ULN ALT in comparison to the current standard Kits, and we recommend it for proper screening of liver disease.

 One of the challenges for the clinical application and interpretation of transaminases is their inconsistent association with the severity of liver damage. Many studies showed that ALT level has poorly predicted the severity of liver injury, and fibrosis progression has poorly correlated with ALT level.^26^ Our results also showed that ALT has low precision in detecting liver disease. However, the trend of ALT precision increased with more severe involvement in grades 2 and 3 of MASLD.

 Respect to the low precision of ALT level, its interpretation could be improved besides the other enzymes like γGT which seems to be a little more sensitive than ALT with a wider range in our results. The best cut-off value remains challenging, and doctors should be informed that higher levels are more specific and require close observation and further investigation.

 Another significant challenge is to apply this new cut-off value, especially when individualized according to gender, in clinical and laboratory settings. Despite the availability of ULN in many research studies, manufacturers’ previous thresholds are still being used for both sexes, even with equal thresholds, without regard for the research results.^27^ The lack of companionship may be due to extremely low cut-off values in many articles. A severe lowering of the threshold in comparison with standard Kits could increase costs without proven benefits, especially due to the low sensitivity of ALT. Severely restricted exclusion of a large number of the population with diabetes, hyperlipidemia, hypertension, overweight, or even patients with higher waist-to-hip ratio or high calorie and carbohydrate diet has been designed in many studies.^9,15,22^ This designation would decrease the cut-off level and its worth and importance due to diminished generalizability to diabetics, overweight, or hyperlipidemic patients.

 On the other hand, defining multiple cut-off values for each subgroup of patients, as computed in some articles,^9^ for example, in different age deciles, patients with diabetes, hyperlipidemia, and obesity, is exhausting and difficult to implement in clinical practice. These different values in each subgroup of patients could be applied only by artificial intelligence in the laboratories and might be more accurate.

 The proven difference in ALT between men and women, as evidenced by all the data, requires eliminating the equal cut-off value for both genders, especially in laboratory clinics. Informing physicians and Laboratory science specialists about this difference according to sex, in addition to introducing the enzyme elevation pattern in particular populations, such as diabetic patients, is the cornerstone of applying the results of research to the real world.

 Only few worthy studies like Korean research by Kang et al in 2011 excluded the patients with abnormal level even by the usual laboratory kits who may involved by any uninvestigated and undiagnosed liver disease^9,22^ and the researchers contintue to use these patients for determining ULN ALT in the distribution curve of “normal population”.On the other hand, an exaggerated policy seems to be placed instead of the exclusion of obscured liver disease and MASLD in many research studies, which is the exclusion of all people with any risk factors of metabolic syndrome due to the high prevalence of MASH especially in the recent years as the main etiology of transaminitis. As mentioned above, this method would exclude a significant portion of the population who need this cut-off value for the interpretation of their liver enzymes and could decrease its generalizability when implemented in the general population, especially in the era of the metabolic syndrome epidemic.

 Preliminary studies for the introduction of ULN ALT from seven decades ago explored the blood donors as a sample of healthy people, and it was believed their transaminase level could be generalized to the community.^3^ Now we understand that at least in our country, many blood donors are athletes and are protein and calorie users for muscle preparation, and they are at risk of MASH and metabolic syndrome. Therefore, blood donors are not proper samples for extracting ULN, which needs to be generalizable to all normal appearing populations.

## Limitations

 The paraclinical findings of this study did not include markers of viral hepatitis, which would have allowed us to eliminate these patients from normal populations; instead, we used only their histories. However, due to the low prevalence of hepatitis in the community,^28^ we estimated that ignoring these patients in a large sample of our normal population without high-risk behaviors has little effect on ULN ALT.

## Conclusion

 The upper cut-off ALT, calculated as the 95th percentile, is 29 U/L in women and 36 U/L in men, which is lower than the cut-off value of the current kits in both sexes. These ULN ALT could be implemented for everybody regardless of involvement by metabolic risk factors.

## Supplementary Files

Supplementary file 1 contains Tables S1 and S2 and Figure S1.

## References

[1] Anderson RA, Lou K, Allen NK (1965). Transaminase levels in a blood donor population. BiblHaematol.

[2] Brandt KH, Meulendijk PN, Poulie NJ, Schalm L, Schulte MJ, Zanen HC. [Value of transaminase determination in donor blood in the prevention of hepatitis through transfusion]. Ned TijdschrGeneeskd 1963;107:2312-9. [Dutch]. 14102353

[3] Prati D, Taioli E, Zanella A, Della Torre E, Butelli S, Del Vecchio E (2002). Updated definitions of healthy ranges for serum alanine aminotransferase levels. Ann Intern Med.

[4] Degertekin B, Tozun N, Demir F, Soylemez G, Yapali S, Bozkurt U (2020). Determination of the upper limits of normal serum alanine aminotransferase (ALT) level in healthy Turkish population. Hepatol Forum.

[5] Huong NT, Karimzadeh S, Thanh NT, Thuan TM, Sabbah GM, Ismaeil K (2022). Updated upper limit of normal for serum alanine aminotransferase value in Vietnamese population. BMJ Open Gastroenterol.

[6] Najmy S, Duseja A, Pal A, Sachdev S, Sharma RR, Marwah N (2019). Redefining the normal values of serum aminotransferases in healthy Indian males. J Clin Exp Hepatol.

[7] Ruhl CE, Everhart JE (2012). Upper limits of normal for alanine aminotransferase activity in the United States population. Hepatology.

[8] Kolahdoozan S, Mirminachi B, Ghajarieh Sepanlou S, Malekzadeh R, Merat S, Poustchi H (2020). Upper normal limits of serum alanine aminotransferase in healthy population: a systematic review. Middle East J Dig Dis.

[9] Kang HS, Um SH, Seo YS, An H, Lee KG, Hyun JJ (2011). Healthy range for serum ALT and the clinical significance of “unhealthy” normal ALT levels in the Korean population. J Gastroenterol Hepatol.

[10] Kim HC, Nam CM, Jee SH, Han KH, Oh DK, Suh I (2004). Normal serum aminotransferase concentration and risk of mortality from liver diseases: prospective cohort study. BMJ.

[11] Khedmat H, Fallahian F, Abolghasemi H, Hajibeigi B, Attarchi Z, Alaeddini F (2007). Serum gamma-glutamyltransferase, alanine aminotransferase, and aspartate aminotransferase activity in Iranian healthy blood donor men. World J Gastroenterol.

[12] Mohamadnejad M, Pourshams A, Malekzadeh R, Mohamadkhani A, Rajabiani A, Ali Asgari A (2003). Healthy ranges of serum alanine aminotransferase levels in Iranian blood donors. World J Gastroenterol.

[13] Kabir A, Pourshams A, Khoshnia M, Malekzadeh F (2013). Normal limit for serum alanine aminotransferase level and distribution of metabolic factors in old population of Kalaleh, Iran. Hepat Mon.

[14] Jamali R, Pourshams A, Amini S, Deyhim MR, Rezvan H, Malekzadeh R (2008). The upper normal limit of serum alanine aminotransferase in Golestan province, northeast Iran. Arch Iran Med.

[15] Akhondi-Meybodi M, Askarzadeh M, Bashardoost N, Amir-Baygi M. Normal range of serum alanine aminotransferase (ALT) levels in healthy population of Yazd and relation to demographic factors. J Shahid Sadoughi Univ Med Sci 2010;18(4):348-54. [Persian].

[16] Idilman IS, Ozdeniz I, Karcaaltincaba M (2016). Hepatic steatosis: etiology, patterns, and quantification. Semin Ultrasound CT MR.

[17] Hernaez R, Lazo M, Bonekamp S, Kamel I, Brancati FL, Guallar E (2011). Diagnostic accuracy and reliability of ultrasonography for the detection of fatty liver: a meta-analysis. Hepatology.

[18] Poustchi H, Eghtesad S, Kamangar F, Etemadi A, Keshtkar AA, Hekmatdoost A (2018). Prospective epidemiological research studies in Iran (the PERSIAN Cohort Study): rationale, objectives, and design. Am J Epidemiol.

[19] Pasdar Y, Najafi F, Moradinazar M, Shakiba E, Karim H, Hamzeh B (2019). Cohort profile: Ravansar Non-Communicable Disease cohort study: the first cohort study in a Kurdish population. Int J Epidemiol.

[20] Zhang P, Wang CY, Li YX, Pan Y, Niu JQ, He SM (2015). Determination of the upper cut-off values of serum alanine aminotransferase and aspartate aminotransferase in Chinese. World J Gastroenterol.

[21] Noureddin M, Lam J, Peterson MR, Middleton M, Hamilton G, Le TA (2013). Utility of magnetic resonance imaging versus histology for quantifying changes in liver fat in nonalcoholic fatty liver disease trials. Hepatology.

[22] Wu WC, Wu CY, Wang YJ, Hung HH, Yang HI, Kao WY (2012). Updated thresholds for serum alanine aminotransferase level in a large-scale population study composed of 34 346 subjects. Aliment PharmacolTher.

[23] Bagheri Lankarani K, Ghaffarpasand F, Mahmoodi M, Lotfi M, Zamiri N, Heydari ST (2013). Non-alcoholic fatty liver disease in southern Iran: a population-based study. Hepat Mon.

[24] Soulier JP. [Measurement of transaminases in blood donors]. Presse Med (1893) 1967;75(12):609-10. [French]. 6019281

[25] Viranuvatti V, Lekayanonda S (1966). Aspartate transaminase (SGO-T) and alanine transaminase (SGP-T) in blood donors, Siriraj Hospital. Am J Proctol.

[26] Ahmed Z, Ahmed U, Walayat S, Ren J, Martin DK, Moole H (2018). Liver function tests in identifying patients with liver disease. Clin Exp Gastroenterol.

[27] Park HN, Sinn DH, Gwak GY, Kim JE, Rhee SY, Eo SJ (2012). Upper normal threshold of serum alanine aminotransferase in identifying individuals at risk for chronic liver disease. Liver Int.

[28] Poorolajal J, Majdzadeh R (2009). Prevalence of chronic hepatitis B infection in Iran: a review article. J Res Med Sci.

