# Supplementary material for: Determining Upper Limit of Alanine Aminotransferase in Iranian Cohort Population Using Ultrasound Screening for Liver Diseases
Source: Middle East J Dig Dis. 2025 Jul 30;17(3):158–66. doi: 10.34172/mejdd.2025.426 (PMC12958307; doi:10.34172/mejdd.2025.426)
Supplement: Supplementary file 1 — contains Tables S1 and S2 and Figure S1. [file mejdd-17-158-s001.pdf]

Middle East J Dig Dis. July 2025;17(3):158-S1

10.34172/mejdd.2025.426

http://mejdd.org

About alcohol consumption, concomittent abnormality in ALT and γGT was found in 120 alcoholic patients and none of them had elevated AST without any abnormality in ALT or γGT. Eleven diabetic patients were found in alcohol consumers with abnormal ALT. So the possible diagnosis in this group may be the combination of Alcoholic steatohepatitis (ASH) and Metabolic dysfunction associated steatohepetitis (MASH) recently known as Metabolic dysfunction and alcohol associated liver disease (MAALSD). Only three patients in this group had positive history of fatty liver (MASLD).
Congestive heart failure assosciated with abnormal ALT was found in 45 patients but only one of them was diagnosed as liver congestion with abnormal alkaline phosphatase. COPD was identified in 11 patients by ICD 10 related codes and only two patients had elevated ALT due to unconfirmed corpulmonale.
Only one patient had ulcerative colitis with normal transaminase level and γGT=37.2 U/l with no documented liver involvement.
Prescribing prednisolone was identified in 188 patients which 61.7% of them had abnormal ALT or γGT (116 patients). Methotrexte was consumed by 42 patients however abnormal ALT or γGT was found only in 3 patients (0.1%). All of these patients were excluded from the target normal population.
Statins were consumed by 870 patients with ALT or γGT elevation in 76 person (7.1%). Statin consumers except those with transaminitis were remained in the normal population to prepare enough generalizability of the resulted ALT range to these patients.

Among 543 screened population with ultra-sonography, seven patients including two men and five women received Prednisolone and/or Methotrexate. Two patients had systemic lupus erythematosus (one of them consumed hydroxychloroquine and the other one had received cyclophosphamide infusion simultaneously), one patient had psoriasis, one involved by lichen planus and the others had Rheumatoid arthritis. Their ALT range was between 28-38 U/l.
One patient who consumed long term antibiotics due to chronic osteomyelitis (following fracture of tibia and fibula) had elevated ALT.

ALT level and its distribution in the cohort population in comparison to the screened individuals of high risk group and ALT level of the remained population according to gender is shown in Figure S1.

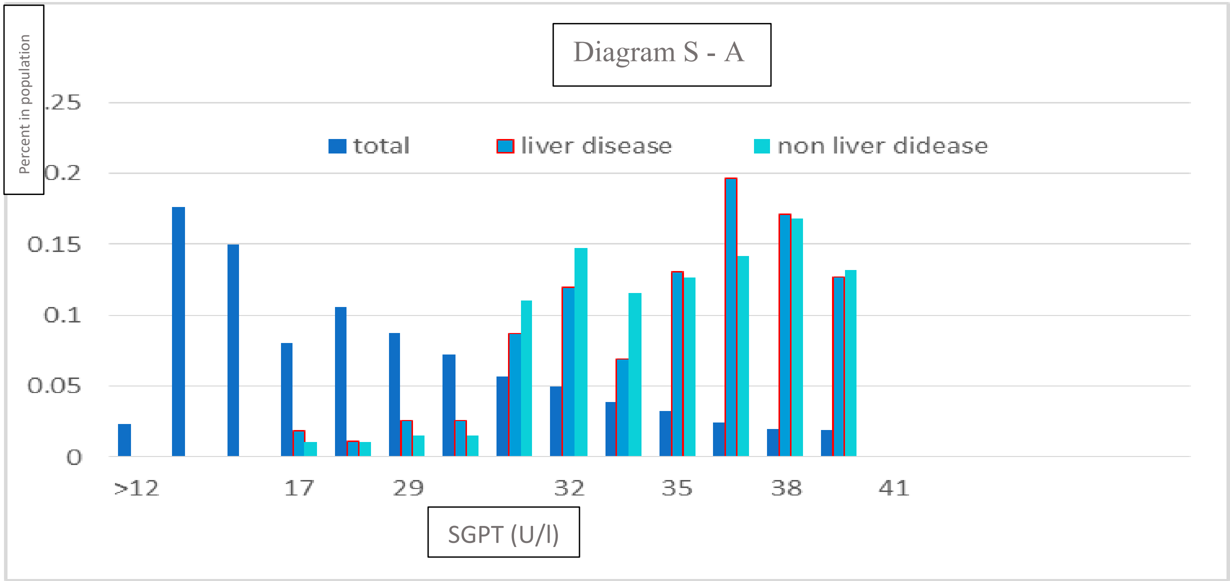

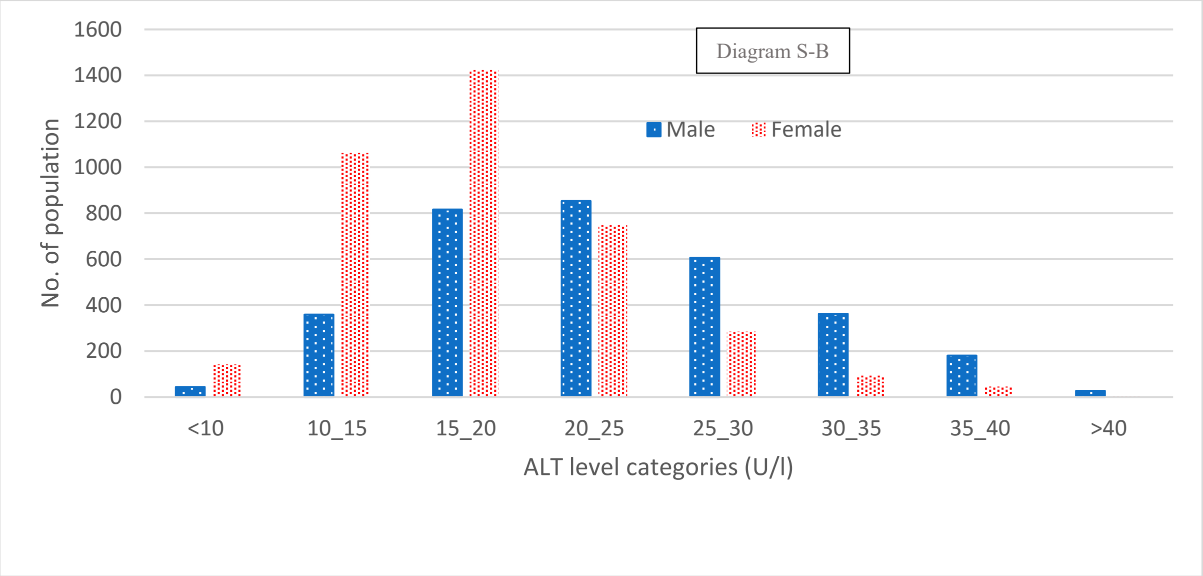

Figure S1: Distribution of ALT level (U/l) in cohort population. Diagram A shows the ALT distribution and its frequencies (in percent) both in total population and screened high risk group according to involvement by liver disease. Diagram B shows the number of individuals in each ALT categories after excluding patients with liver disease.

Table S1: Comparison between ALT and GGT trend according to grading of fatty liver in ultra-sonography

Variables	**Grade**	**Median (Min-Max)**	**Percentile 50 (95%CI)**	**P Value**
SGPT	Not fatty	34.8(18_40.9)	34.8 (21.9_40.3)	0.2
	Grade1	34.7(17.6_40.9)	34.7 (25.5_40.7)	
	Grade2	35.5(19.1_40.9)	35.5 (24.0_40.1)	
	Grade3	34.05(28.9_39.9)	34.0 (28.9_39.9)	
GGT	Not fatty	25.6(6_141.8)	25.6 (12.3_73.0)	0.1
	Grade1	28.6(8.3_248.9)	28.6 (14.6_80.7)	
	Grade2	29.3(13.5_95.9)	29.3 (16.3_62.6)	
	Grade3	33.25(18.5_78.92)	33.2 (18.5_78.9)	

Table S2: Cut points analysis of ALT for sensetivity, specifity and positive and negative likelihood ratio: Part A shows analysis of best point for SGOT, SGPT, ALP and γGT by Area under the Receiver Operating Characteristic (AUROC) curve. Part B shows sensitivity and specificity analysis of 95th percentile cut off point.

**A.** By ROC curve		**Cut off**	**Roc area**	**Sensitivity**	**Specificity**	**LR+**	**LR-**
**Male**	**SGOT**	23.3	65.16	58.22	64.56	1.64	0.67
	**SGPT**	28.9	73.48	71.64	66.90	2.16	0.42
	**γGT**	27	69.52	61.07	66.79	1.38	0.58
	**ALP**	192.7	53.45	51.01	50.51	1.03	0.96
**Female**	**SGOT**	19.3	56.92	51.08	60.96	1.30	0.80
	**SGPT**	19.7	65.94	60.73	61.97	1.59	0.63
	**γGT**	20.5	63.27	50.40	68.06	1.57	0.72
	**ALP**	191.0	54.30	51.42	53.86	1.11	0.90
**Total**	**SGOT**	20.1	57.5	55.87	55.36	1.25	0.79
	**SGPT**	22.8	63.55	60.23	60.57	1.57	0.65
	**γGT**	21.2	62.08	58.14	59.30	1.42	0.70

	**ALP**	191.2	52.57	51.96	51.05	1.06	0.94
**B.** 95th percentile		**Cut off**	**Roc area**	**Sensitivity**	**Specificity**	**LR+**	**LR-**
**Male**	**SGOT**	29.0	85.16	12.42	95.77	2.93	0.91
	**SGPT**	36.1	78.55	52.85	82.30	2.98	0.57
	**γGT**	35.6	77.36	41.44	82.60	2.38	0.70
	**ALP**	267.2	81.10	12.92	91.04	1.44	0.95
**Female**	**SGOT**	26.5	78.50	17.93	90.98	1.98	0.90
	**SGPT**	28.8	79.63	27.70	90.32	2.88	0.79
	**γGT**	31.7	77.43	23.61	88.52	2.05	0.86
	**ALP**	275.0	76.60	12.15	89.88	1.19	0.9
**Total**	**SGOT**	28.0	79.74	20.45	90.21	2.08	0.88
	**SGPT**	33.8	78.55	33.24	86.55	2.47	0.77
	**γGT**	33.9	77.15	29.93	85.50	2.06	0.81
	**ALP**	271.4	78.85	12.46	90.56	1.33	0.96

The strong advantages of our study consist of: 1) Selecting large sample of normal cohort population, especially by providing the exclusion of liver disease by US in high risk patients, 2) Lack of excluding patients with metabolic risk factors and 3) Eliminating un-investigated patients with abnormal liver enzymes from the distribution diagram of normal population. This designation prepare a generalizable cut-off value of ALT for all patients including those with metabolic risk factors like diabetes, hyperlipidemia or obesity.

Other limitations:

We used a sample of high risk population for US screening because performing US in all cohort participants was impractical.

In order to exclude liverdisease, individuals were screened only based on the ICD-10 codes of different type of liver disease, liver enzymes, history of liver disease or liver related diseases compatible with the exclusion criteria.

Liver histology as the gold standard to identify liver disease could not be considered at all in normal general population.

More researches especially in different regions of Iran without exclusion of metabolic risk factors could improved the ALT occuracy for clinical practice.
